# Analysis of characteristics of hospitalized patients with chronic insomnia: a single-center retrospective study

**DOI:** 10.3389/fpsyt.2026.1682122

**Published:** 2026-04-21

**Authors:** Hong Su, Jiayu Bai, Shaoyong Liang, Zongding Wang

**Affiliations:** 1 Department of Hepatobiliary Surgery, Fengjie County People’s Hospital of Chongqing, Chongqing, China; 2 Department of Hepatobiliary Surgery, Fengjie Hospital, The Second Affiliated Hospital of Chongqing Medical University, Chongqing, China

**Keywords:** chronic insomnia, dyslipidemia, hospitalized patients, main diagnosis, tumor

## Abstract

**Objective:**

Insomnia is a prevalent health issue within the general population. Nonetheless, there is a paucity of research specifically addressing chronic insomnia among hospitalized patients. Consequently, the objective of this study is to investigate chronic insomnia in adult inpatients.

**Methods:**

A retrospective analysis was conducted on hospitalized patients at Fengjie County People’s Hospital in Chongqing from January 2022 to June 2025. The study included patients aged 18 to 100 years, with comprehensive documentation of their demographic information, laboratory test results, and insomnia treatment details. Patients with incomplete data or those under 18 years of age were excluded from the study. The analysis focused on the age distribution, gender ratio, and BMI distribution of the patients, as well as the prevalence of primary diagnostic categories and the distribution characteristics of fasting blood glucose levels and dyslipidemia.

**Results:**

The study included a total of 871 patients, with a male representation of 39.6%. The mean body mass index (BMI) was 23.1 ± 3.7 kg/m², and the mean age was 64.1 ± 13.9 years. The predominant sources of disease were identified as infectious diseases, cardiovascular diseases, neurological disorders, tumors, and musculoskeletal conditions. A significant proportion of patients presented with elevated fasting blood glucose levels and dyslipidemia.

**Conclusion:**

Chronic insomnia in hospitalized patients predominantly affects elderly women. Chronic insomnia in hospitalized patients predominantly affects elderly women, who primarily present with infectious diseases, cardiovascular conditions, neurological disorders, tumors, and musculoskeletal issues. These patients often exhibit dyslipidemia and elevated fasting blood glucose levels, necessitating clinical attention.

## Introduction

Insomnia represents a significant threat to human health and is characterized by a high global prevalence (1). Individuals affected by insomnia often encounter difficulties in initiating sleep, maintaining sleep, and achieving restorative sleep, which results in substantial daytime impairments such as persistent fatigue, reduced concentration, and mood disturbances, including irritability and anxiety. In severe instances, insomnia can precipitate a range of physical and mental health disorders, thereby eliciting increased concern among clinicians and researchers (2). Insomnia is categorized based on its duration into acute and chronic forms. Chronic insomnia is particularly marked by challenges in initiating sleep, maintaining sleep, experiencing early morning awakenings, or enduring poor sleep quality. Importantly, at least one of these disturbances must occur at least three times per week and persist for a minimum of three months to meet the diagnostic criteria for chronic insomnia (3).

According to the extant literature, a variety of medical conditions can precipitate insomnia disorders (4). In severe instances, these conditions may progress to chronic insomnia. Such conditions include tumors (5), chronic pain (6), hyperthyroidism (7), sleep apnea syndrome (8), and post-traumatic stress disorder (9), among others. Additionally, several mental disorders, such as major depressive disorder and schizophrenia, are implicated (10–12). The symptoms or pathological processes associated with these physical and mental disorders can directly disrupt sleep, thereby contributing to the onset or persistence of chronic insomnia. The chronic insomnia experienced by hospitalized patients is often associated with their underlying medical conditions, primarily manifesting as secondary insomnia. This contrasts with insomnia observed in community-dwelling individuals, which is frequently attributed to factors such as occupational, personal, or academic stress, lifestyle choices, familial or emotional issues, and environmental influences, and is predominantly classified as primary insomnia (4). Regarding severity, insomnia among community patients tends to be mild to moderate, whereas hospitalized patients typically experience more severe and complex forms. There exists a bidirectional relationship between chronic insomnia and physical or mental illnesses, wherein each exacerbates the other, forming a “vicious cycle.” For example, chronic insomnia can exacerbate anxiety, which subsequently hinders the ability to fall asleep, thereby worsening insomnia and further intensifying anxiety (13). This cyclical interaction significantly complicates treatment efforts. Addressing sleep disturbances or treating physical and mental illnesses in isolation often results in suboptimal outcomes. Recognizing the bidirectional relationship is essential. Clinical interventions should employ a “synergistic treatment” approach to optimize therapeutic outcomes (14).

Currently, the management of chronic insomnia primarily involves a multifaceted approach, incorporating pharmacological interventions, psychotherapy, and physical therapy (15). Pharmacological treatments predominantly include non-benzodiazepine medications, benzodiazepines, and traditional Chinese medicine. In the context of hospitalized patients, pharmacotherapy remains the principal treatment modality for chronic insomnia. Currently, there is a paucity of research concerning the underlying diseases, demographic characteristics, and associated clinical features of hospitalized patients with chronic insomnia. Consequently, this study undertakes a retrospective analysis of these aspects at our single-center hospital over recent years, and provides a preliminary examination.

## Methods

### Study population

The study population comprised adult inpatients who received treatment at Fengjie County People’s Hospital in Chongqing between January 2022 and June 2025. Clinical data and outcomes were retrospectively extracted from electronic medical records by two members of the research team and subsequently verified by an additional two members (Based on the discharge diagnosis of ICD-10 in the electronic medical record system, the data of patients who were diagnosed with chronic insomnia and whose insomnia had been recorded for more than 3 months in the medical records were collected). The dataset included 1,497 individuals, ranging in age from 18 to 100 years, all diagnosed with chronic insomnia. Data collection encompassed variables such as gender, age, alcohol consumption, laboratory test results (including blood routine, liver and kidney function, and lipid levels), and medication history. Alcohol consumption history was categorized as any prior alcohol use, with intake defined as exceeding 210 grams per week for men and 140 grams per week for women(Having consumed alcohol continuously for more than one year and not quitting before being hospitalized.). Chronic hepatitis B was identified by a diagnosis of hepatitis B, while a history of cancer was characterized by a previous diagnosis and treatment of malignant tumors or current treatment for such conditions(lung cancer, gastrointestinal cancer, breast cancer, head and neck cancer, and other malignant neoplasms). A history of insomnia treatment was defined as the use of oral medications for insomnia for a duration exceeding one month. The use of oral lipid-lowering medications for a duration exceeding one month was classified as having a history of lipid-lowering treatment. Participants with incomplete or missing laboratory data were excluded from the analysis. Consequently, the study ultimately comprised 871 participants.

### Laboratory measurements

The patients’ height and weight were documented, and their Body Mass Index (BMI) was calculated by dividing the weight by the square of the height. Data from the initial tests conducted during the patients’ hospitalization were collected, encompassing various parameters: hematological parameters including white blood cells (WBC), hemoglobin (Hb), and platelets (PLT); inflammatory markers such as C-reactive protein (CRP); liver function indicators including total bilirubin (T-Bil), alanine aminotransferase (ALT), aspartate aminotransferase (AST), γ-glutamyl transferase (GGT), and albumin (ALB); renal function markers such as blood urea nitrogen (BUN) and creatinine (Cre); lipid profile parameters including total cholesterol (TC), triglycerides (TG), high-density lipoprotein cholesterol (HDL-C), and low-density lipoprotein cholesterol (LDL-C); coagulation-related factors such as D-dimer; and fasting blood glucose (FBG). FBG refers to the blood sugar level measured after fasting for more than 8 to 12 hours; dyslipidemia is defined as having total cholesterol or triglycerides, low-density lipoprotein cholesterol above the normal reference range, while high-density lipoprotein cholesterol below the normal reference range; the BMI range is classified according to the general standards.

### Statistical analysis

In the analysis of categorical variables within the clinical dataset, frequency and percentage statistics were utilized. For continuous variables adhering to a normal distribution, the mean ± standard deviation was used for representation. Conversely, for continuous variables not conforming to a normal distribution, the median was employed. When comparing two groups, if the data exhibit a normal distribution with homogeneous variances, an independent sample t-test is appropriate; however, if variances are heterogeneous, the Welch t-test is recommended. For non-normally distributed data, the Mann-Whitney U test is applied. The comparison of measurement data among multiple groups was conducted using one-way analysis of variance (ANOVA). If the data did not meet the conditions of normal distribution or homogeneity of variance, the Kruskal-Wallis H test was used. Pairwise comparisons between groups were performed using the Tukey method. At the same time, the Bonferroni method was employed to correct for the inflation of type I error caused by multiple comparisons to control the overall test level. The two-sided significance level α = 0.05, and P < 0.05 was considered statistically significant.

## Results

### Clinical characteristics

A total of 871 hospitalized patients diagnosed with chronic insomnia were enrolled in this study. Among these individuals, 39.6% were male, with a mean age of 64.1 years (± 13.9) and a mean body mass index (BMI) of 23.1 (± 3.7). The prevalence of malignant tumors within this population was 10.45%, while 3.56% reported a history of alcohol consumption and 2.84% had a history of hepatitis B. Notably, 84.96% of the patients with chronic insomnia received pharmacological treatment for a duration of at least one month. The average lipid profiles of these patients indicated elevated levels, with total cholesterol (TC) averaging 1.8 (± 3.8)mmol/L, triglycerides (TG) averaging 4.6 (± 1.2)mmol/L, high-density lipoprotein cholesterol (HDL-C) averaging 1.2 (± 0.4)mmol/L, and low-density lipoprotein cholesterol (LDL-C) averaging 2.8 (± 1.0)mmol/L. Furthermore, 26.18% of the patients were undergoing lipid-lowering therapy. The average fasting blood glucose level was recorded at 7.5 (± 6.1)mmol/L, suggesting a state of hyperglycemia, and the average D-dimer level was elevated, averaging 1.8 (± 3.5)mg/L. Importantly, all inflammatory markers, complete blood count parameters, and indicators of liver and kidney function remained within normal limits. Detailed information regarding these indicators is presented in **Table 1**.

**Table 1:** Baseline characteristics of individuals.

Variables	N = 871
Age (years)	64.1 ± 13.9
BMI (kg/m^2^)	23.1 ± 3.7
Male (%)	39.6
WBC (10^9^/L)	6.9 ± 2.9
Hb (g/L)	128.8 ± 44.6
PLT (10^9^/L)	202.5 ± 76.8
CRP (mg/L)	18.5 ± 153.2
T-Bil (μmol/L)	15.3 ± 19.2
ALT (U/L)	31.8 ± 118.9
AST (U/L)	35.8 ± 117.6
GGT (U/L)	45.8 ± 65.9
ALB(g/L)	40.6 ± 6.7
BUN(mmol/L)	6.8 ± 4.1
Cre(μmol/L)	73.5 ± 52.1
TC (mmol/L)	1.8 ± 3.8
TG (mmol/L)	4.6 ± 1.2
HDL-C (mmol/L)	1.2 ± 0.4
LDL-C (mmol/L)	2.8 ± 1.0
D-dimer(mg/L)	1.8 ± 3.5
FBG(mmol/L)	7.5 ± 6.1
Malignant tumors(%)	10.45
Alcohol use(%)	3.56
Insomnia treatment(%)	84.96
Hepatitis B virus (HBV) infection(%)	2.41
Lipid-lowering drugs(%)	26.18

WBC, White Blood Cell Count; Hb, Hemoglobin; PLT, Platelet Count; CRP, C-Reactive Protein; T-Bil, Total Bilirubin; ALT, Alanine Aminotransferase; AST, Aspartate Aminotransferase; GGT, Gamma-Glutamyl Transferase; ALB, Albumin; BUN, Blood Urea Nitrogen; Cre, Creatinine; TC, Total Cholesterol; TG, Triglyceride; HDL-C, High-Density Lipoprotein Cholesterol; LDL-C, Low-Density Lipoprotein Cholesterol; D-dimer, D-dimer; FBG, Fasting Blood Glucose.

### The distribution of diseases associated with chronic insomnia

**Figure 1:**
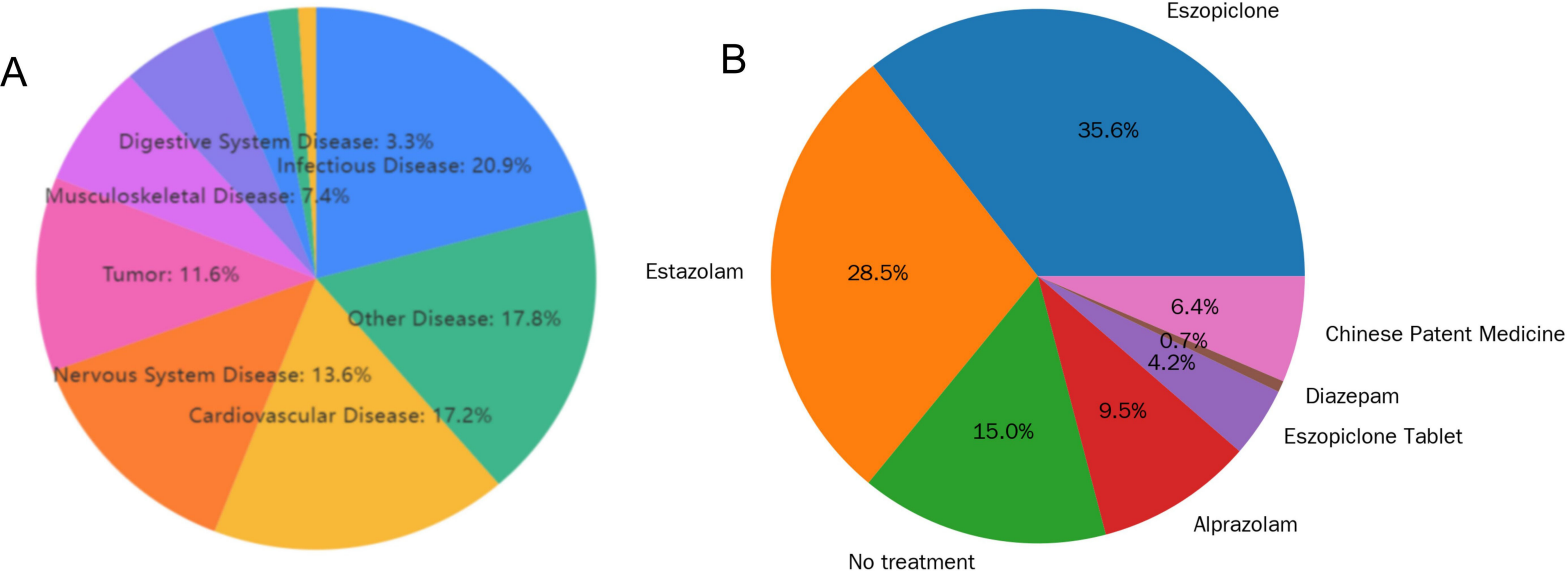
The main diagnoses and the medications used for treating insomnia in patients with chronic insomnia. **(A)** Pie chart showing the proportion of the main diagnostic sources for hospitalized patients with chronic insomnia. **(B)** Medication usage for chronic insomnia treatment.

The distribution of diseases associated with chronic insomnia was subjected to statistical analysis, categorized by the underlying sources of the diseases. Infectious diseases constituted the largest category, accounting for 20.9% of cases, suggesting that infectious diseases are relatively prevalent among hospitalized patients with chronic insomnia. Cardiovascular diseases represented 17.2% of the cases, indicating a significant association between cardiovascular conditions and chronic insomnia within this patient population. Other diseases, encompassing a variety of conditions not classified elsewhere, comprised 17.8% of the cases. Nervous system diseases accounted for 13.6%, highlighting the potential bidirectional relationship between neurological conditions and sleep disturbances. Finally, tumors were present in 11.6% of the cases, reflecting the propensity for patients with tumors to experience chronic insomnia due to both the disease and its treatment. Musculoskeletal diseases, which affect approximately 7.4% of the population, can significantly impair sleep quality, potentially resulting in chronic insomnia. This relationship is illustrated in **Figure 1A**.

### Classification of drug treatments for insomnia patients

Eszopiclone is the most frequently utilized medication, with a usage rate of 35.6%, suggesting its prevalent application in the treatment of chronic insomnia within this patient cohort. Estazolam follows with a usage rate of 28.5%, indicating its widespread use as a benzodiazepine in therapeutic interventions. Alprazolam, another benzodiazepine, accounts for 9.5% of usage, demonstrating its role in treatment. Eszopiclone tablets specifically represent 4.2% of the usage. Diazepam, also a benzodiazepine, has a relatively low usage rate of 0.7% in this group. Chinese patent medicines constitute 6.4% of the treatment approaches, highlighting the integration of traditional medicine in managing chronic insomnia and reflecting the diversity of therapeutic strategies. Notably, 15.0% of the patients did not receive any pharmacological intervention, indicating a subset of hospitalized patients with chronic insomnia who were not subjected to drug treatment. The distribution of drug treatment for chronic insomnia is shown in **Figure 1B**.

### Characteristics of chronic insomnia in different age groups

**Figure 2:**
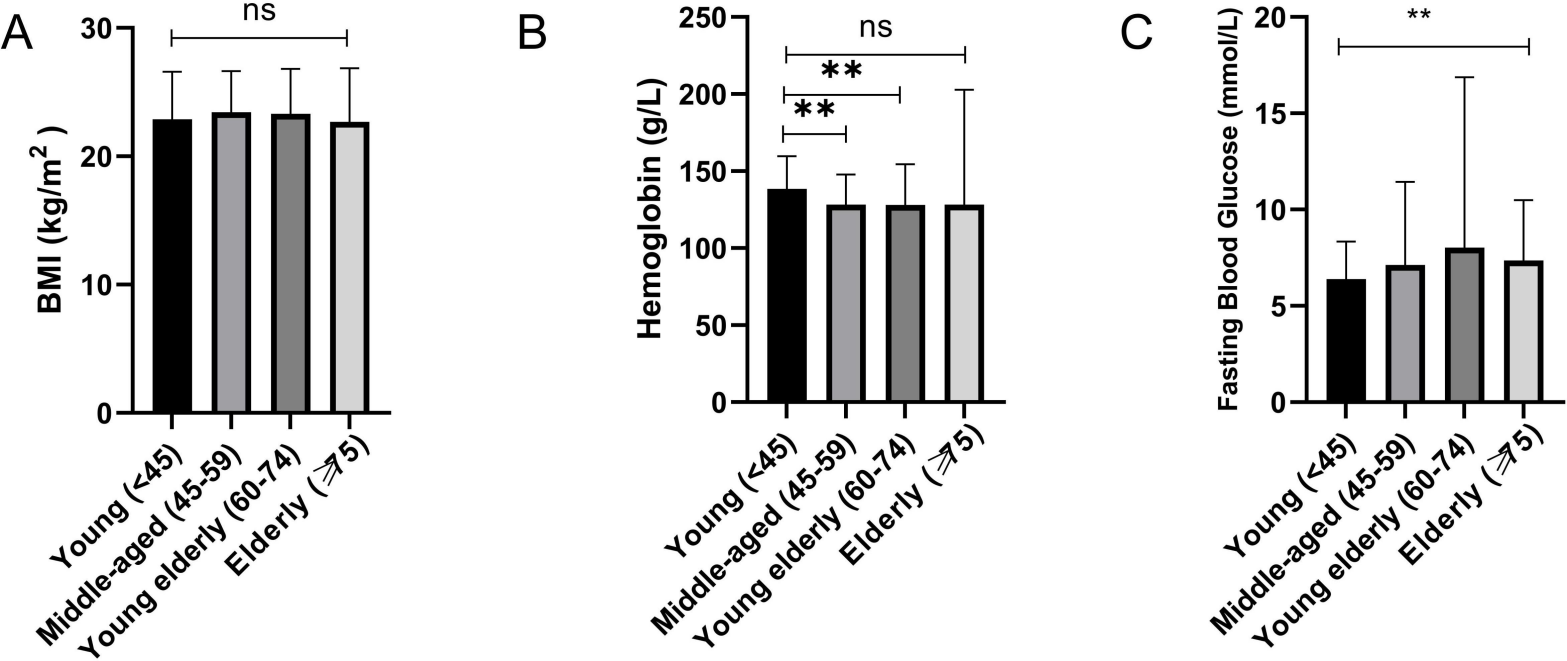
Comparison of BMI, hemoglobin and fasting blood glucose among different age groups of patients with chronic insomnia. **(A)** Comparison results of BMI among different age groups. **(B)** Comparison of hemoglobin levels among different age groups. **(C)** Comparison results of fasting blood glucose among different age groups. (ns:P>0.05; **P<0.01).

The study categorizes participants into four age groups: young individuals (< 45 years, n=53), middle-aged individuals (45-59 years, n=262), young elderly individuals (60-74 years, n=308), and elderly individuals (≥ 75 years, n=248). The overall sample size increases with age, indicating a higher prevalence of chronic insomnia among middle-aged and elderly populations. In terms of Body Mass Index (BMI), the middle-aged cohort (23.45 ± 3.23) exhibited slightly higher values compared to the young (22.9 ± 3.68) and young elderly groups (23.30 ± 3.50), whereas the elderly group had a marginally lower BMI (22.68 ± 4.19). Despite these variations, all groups maintained BMI values within the normal range, with individual differences observed across age categories (**Figure 2A**). Hemoglobin levels were highest in the young group (138.36 ± 21.39 g/L) and showed a decline in the middle-aged (128.30 ± 19.42 g/L), young elderly (127.97 ± 26.53 g/L), and elderly groups (128.25 ± 74.61 g/L), with significant individual variability noted in the elderly cohort (**Figure 2B**). Fasting blood glucose levels demonstrated an age-related increase, with the young group (6.39 ± 1.95 mmol/L) displaying slightly elevated levels, followed by progressive increases in the middle-aged (7.13 ± 4.31 mmol/L), young elderly (8.02 ± 8.86 mmol/L), and elderly groups (7.36 ± 3.13 mmol/L).Despite a decrease in the elderly cohort, their levels remained atypical(**Figure 2C**). The younger subset within the elderly population exhibited the most significant fluctuations, underscoring a pronounced risk of hyperglycemia, as illustrated in **Table 2**.

**Table 2:** Distribution characteristics of BMI, hemoglobin, and fasting blood glucose.

Age group	Sample size	BMI (mean ± SD)	Hemoglobin (g/L)	Fasting blood glucose (mmol/L)
Young (<45)	53	22.9 ± 3.68	138.36 ± 21.39	6.39 ± 1.95
Middle-aged (45-59)	262	23.45 ± 3.23	128.30 ± 19.42	7.13 ± 4.31
Young elderly (60-74)	308	23.30 ± 3.50	127.97 ± 26.53	8.02 ± 8.86
Elderly (≥75)	248	22.68 ± 4.19	128.25 ± 74.61	7.36 ± 3.13

### BMI and age group characteristics

In patients diagnosed with chronic insomnia, the distribution of body mass index (BMI) categories is as follows: individuals with a normal weight constitute the largest proportion, comprising 58.9% (n = 558), suggesting that the majority of chronic insomnia patients maintain a BMI within the normal range. Meanwhile, 22.1% (n = 209) are classified as overweight, 7.6% (n = 72) as underweight, and 3.4% (n = 32) as obese, indicating the presence of atypical weight conditions among a subset of these patients. As shown in **Table 3**.

**Table 3:** Distribution characteristics of BMI.

BMI classification	Sample size	BMI (mean ± SD)
Underweight	72(7.6%)	16.86 ± 2.32
Normal	558(58.9%)	22.03 ± 1.73
Overweight	209(22.1%)	26.92 ± 1.31
Obese	32(3.4%)	31.96 ± 2.47

The distribution of chronic insomnia patients across various age groups within different BMI categories (underweight, normal weight, overweight, obese) was examined, focusing on sample size and proportional representation. The analysis of the correlation between age and BMI classification revealed that, among chronic insomnia patients, the normal weight category was the most prevalent (58.9%). However, variations in BMI distribution were observed across different age groups: the young cohort exhibited a notable proportion of underweight individuals and a comparatively low proportion of obesity; the middle-aged cohort demonstrated the highest proportion of individuals with normal weight and a relatively elevated proportion of obesity; the young elderly cohort had the highest proportion of overweight individuals; and the elderly cohort showed a relatively high proportion of underweight individuals. These findings are illustrated in **Table 4**.

**Table 4:** Age - BMI cross - tabulation distribution (sample size and proportion).

Age group	Underweight	Normal	Overweight	Obese
Young (<45)	8 (15.1%)	31 (58.5%)	14 (26.4%)	0 (0.0%)
Middle-aged (45-59)	14 (5.3%)	174 (66.4%)	62 (23.7%)	12 (4.6%)
Young elderly (60-74)	22 (7.1%)	192 (62.3%)	84 (27.3%)	10 (3.2%)
Elderly (≥75)	28 (11.3%)	161 (64.9%)	49 (19.8%)	10 (4.0%)
Total	72	558	209	32

### The distribution characteristics of dyslipidemia in patients with insomnia

**Figure 3:**
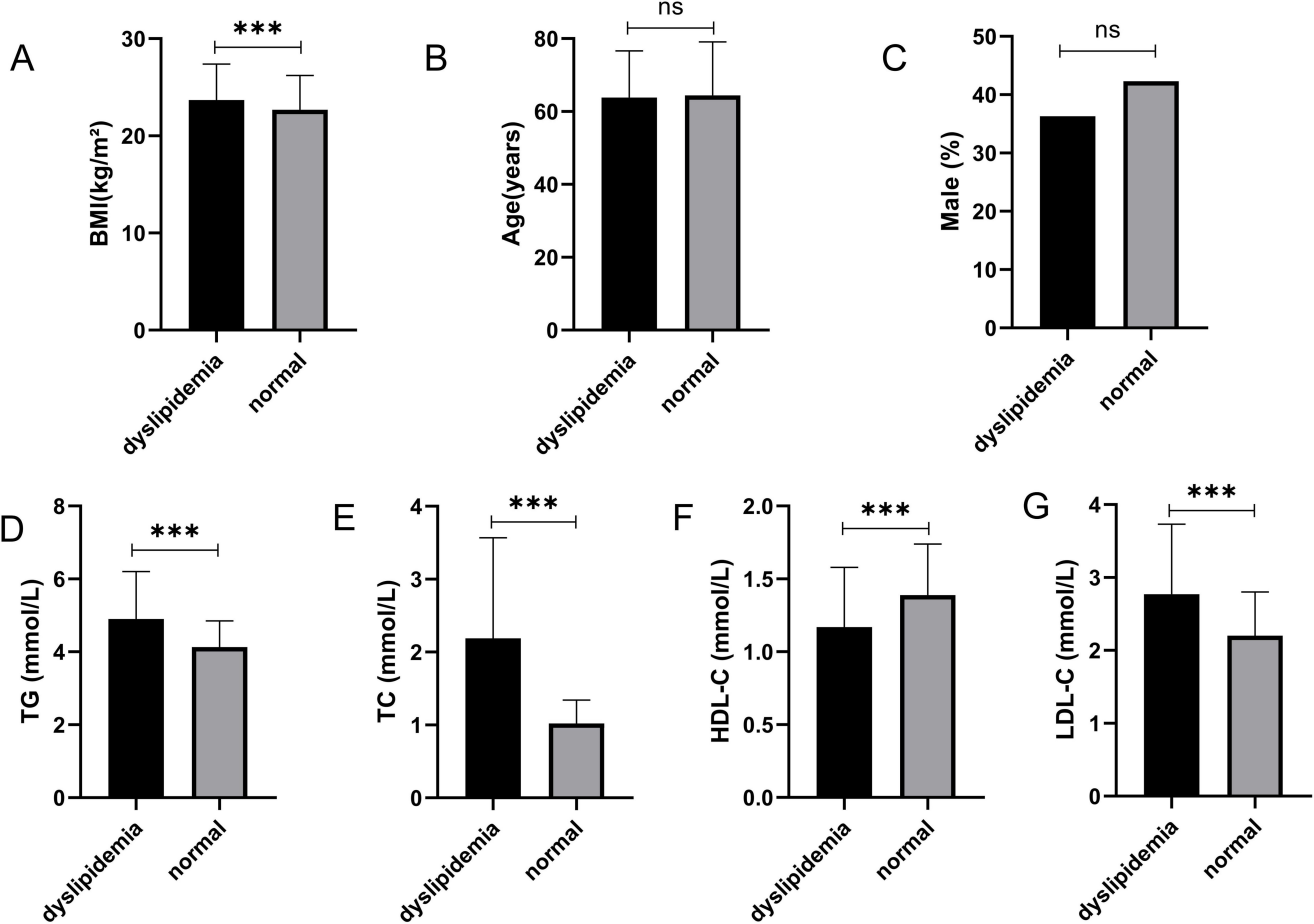
Comparison between patients with dyslipidemia and those with normal blood lipid levels among patients with chronic insomnia. **(A)** Comparison of BMI between patients with normal blood lipid levels and those with dyslipidemia. The BMI of the dyslipidemia group was significantly higher than that of the normal group. **(B)** Comparison of age between patients with normal blood lipid levels and those with dyslipidemia. **(C)** Comparison of the proportion of males between patients with normal blood lipid levels and those with dyslipidemia. **(D–G)** Comparison of TG, TC, HDL - C, and LDL - C levels between the dyslipidemia group and the normal group. (ns:P>0.05; ***P<0.001).

Based on age group classification, the prevalence of dyslipidemia is distributed as follows: In the young cohort (< 45 years, n=59), the overall prevalence of dyslipidemia is 32.19% (n=19), representing the lowest rate among all age groups. Within this group, the highest incidence is observed in abnormal HDL levels (18.64%), followed by TG abnormalities (10.17%), whereas the prevalence of abnormal TC and LDL levels is minimal, with only one individual affected in each category (1.69%). In the middle-aged cohort (45-59 years, n=274), the overall prevalence of dyslipidemia rises to 56.94% (n=156), the highest among the groups. The incidences of TG (21.17%) and HDL (21.90%) abnormalities are both substantial and comparable, while the prevalence of abnormal TC (7.30%) and LDL (6.57%) levels is notably higher than in the young group. In the young elderly cohort (60-74 years, n=317), the overall prevalence of dyslipidemia is 47.63% (n=151), which is lower than that of the middle-aged group. HDL abnormalities (20.50%) are the most prevalent, followed by TG abnormalities (17.67%), whereas the proportions of abnormal TC and LDL levels are reduced compared to the middle-aged group. In the elderly cohort, defined as individuals aged 75 years and older (n = 221), the overall prevalence of dyslipidemia was 33.47% (n = 74), a rate comparable to that observed in the younger cohort. Within this group, the incidence of abnormal lipid indicators was generally low. However, the prevalence of high-density lipoprotein (HDL) abnormalities was the most pronounced at 17.19%, followed by triglycerides (TG) at 9.95%. In contrast, the prevalence of total cholesterol (TC) and low-density lipoprotein (LDL) abnormalities was below 4% (**Figure 3**). Notably, the middle-aged cohort exhibited the highest overall prevalence of dyslipidemia, with HDL abnormalities being a significant concern across all age groups. As shown in **Table 5**.

**Table 5:** Dyslipidemia and its distribution among insomniac patients.

Age group	Total number	TC	TG	HDL	LDL	Dyslipidemia
Young (<45)	59	1 (1.69%)	6 (10.17%)	11 (18.64%)	1 (1.69%)	19 (32.19%)
Middle - aged (45 - 59)	274	20 (7.30%)	58 (21.17%)	60 (21.90%)	18 (6.57%)	156 (56.94%)
Young elderly (60 - 74)	317	19 (5.99%)	56 (17.67%)	65 (20.50%)	11 (3.47%)	151 (47.63%)
Elderly (≥75)	221	8 (3.62%)	22 (9.95%)	38 (17.19%)	6 (2.71%)	74 (33.47%)

### Distribution of lipid-lowering drug usage

The study examined the utilization of lipid-lowering medications among patients with chronic insomnia, comparing those with abnormal lipid profiles to those with normal lipid levels. The findings indicated that, with the exception of HDL-C, the prevalence of lipid-lowering drug use was significantly higher among individuals with abnormal triglycerides (TG), total cholesterol (TC), and low-density lipoprotein cholesterol (LDL-C) compared to the normal lipid group. Notably, the group with abnormal LDL-C exhibited the highest usage rate at 41.2%, followed by the group with abnormal TG at 38.2%. Conversely, the group with abnormal HDL-C had a lower usage rate of 23.2% compared to the normal group, which had a rate of 30.8%. Overall, the data demonstrate a significantly increased use of lipid-lowering drugs among individuals with lipid abnormalities, particularly those with LDL-C and TG irregularities, as detailed in **Table 6**.

**Table 6:** Abnormal and normal lipids and lipid - lowering drug use.

Indicator (mmol/L)	Dyslipidemia	Lipid - lowering drugs	Normal	Lipid - lowering drugs
TG	420 (2.19 ± 1.38)	38.2%	238 (1.02 ± 0.32)	26.7%
TC	440 (4.90 ± 1.30)	37.4%	219 (4.13 ± 0.72)	27.5%
HDL - C	341 (1.17 ± 0.41)	23.2%	530 (1.39 ± 0.35)	30.8%
LDL - C	557 (2.77 ± 0.96)	41.2%	102 (2.20 ± 0.60)	28.9%

## Discussion

Despite the high prevalence of insomnia, there is a paucity of research concerning the insomnia status of hospitalized patients. Through a retrospective analysis of adult inpatients with chronic insomnia, we identified several noteworthy findings. Among the patients with chronic insomnia, 60.4% were female, a proportion significantly higher than that of male patients. In terms of age distribution, the majority of patients were over 45 years old, with those under 45 being notably rare. The average age of the cohort was 64.1 ± 13.9 years. Research indicates that the prevalence of insomnia differs across various demographic groups and tends to rise with advancing age, with elderly individuals being particularly susceptible (16). Additionally, the incidence of insomnia is generally higher in women compared to men (17). The symptoms and underlying causes of insomnia also differ across age groups (1). These findings align with existing literature on the subject. Most patients with insomnia are treated with oral medications, primarily benzodiazepines, while a minority receive traditional Chinese medicine. A small proportion of patients do not undergo any form of treatment.

The treatment for insomnia varies depending on the underlying cause. For hospitalized patients, insomnia is mostly secondary insomnia. The data we reviewed retrospectively pertains to all patients in the hospital, and they have a wide variety of accompanying diseases, making the treatment complex and diverse. This is quite different from primary insomnia. Therefore, the intervention methods should be different.

Regarding the observation that 15.0% of patients with chronic insomnia did not receive pharmacological treatment, several potential factors may explain this phenomenon, with important implications for clinical practice. First, patient preferences and concerns about medication side effects (e.g., daytime drowsiness, dependency, or cognitive impairment associated with benzodiazepines) could lead to the refusal or avoidance of drug therapy (18). Second, some patients may have had contraindications to conventional insomnia medications, such as a history of substance abuse, severe liver or kidney dysfunction. Third, access to non-pharmacological interventions, such as cognitive behavioral therapy for insomnia (CBT-I), may have influenced clinical decision-making—CBT-I is recommended as a first-line treatment for chronic insomnia by clinical guidelines, and its availability could lead to the prioritization of non-drug approaches. Although this group of patients is not extracted from the medical record system (19). Additionally, clinical judgments based on disease severity and comorbidities likely played a role: patients with mild insomnia or those with well-controlled comorbid conditions (e.g., stable cardiovascular disease or mild neurological disorders) may have been deemed suitable for non-pharmacological management alone. Conversely, some elderly patients or those with advanced comorbidities may have had limited tolerance to medications, leading clinicians to opt for conservative treatment strategies. Understanding these factors is clinically relevant as it highlights the need for personalized treatment plans that align with patient characteristics, preferences, and clinical contexts, emphasizing the importance of a holistic approach to insomnia management in hospitalized populations.

We conducted a comprehensive analysis using the height and weight index. Examination of data across various age groups revealed that the Body Mass Index (BMI) of individuals aged 45 to 74 was marginally higher compared to other age groups, although this difference lacked statistical significance. Upon evaluating BMI according to classification criteria, it was observed that 58.9% of the population fell within the normal range, while the combined prevalence of overweight and obesity was approximately 25.5%, with the remainder classified as underweight. Cross-analysis of age groups and BMI indicated variability in BMI distribution across different age cohorts. Notably, the prevalence of overweight individuals was highest within the 60-74 age group.

In this study, we conducted an analysis of the primary diagnoses among patients suffering from chronic insomnia. Utilizing a disease system classification framework, our findings indicate that infectious diseases, cardiovascular system diseases, and neurological disorders constitute the largest proportions of diagnoses, followed by neoplasms and musculoskeletal disorders. Notably, infectious diseases represented the highest proportion. Cardiovascular and neurological diseases followed in prevalence. This distribution contrasts with the current understanding of insomnia in non-hospitalized populations, where neurological disorders, mental health disorders, and endocrine system diseases are most commonly associated with insomnia, according to recent literature (20). The observed discrepancy may be attributed to the propensity for infections to precipitate insomnia, while hospitalized patients with cardiovascular and neurological conditions may experience or continue to experience insomnia due to physical discomfort and psychological stress (21).Neoplastic disorders and conditions affecting the musculoskeletal system are often associated with chronic pain, which can lead to persistent insomnia. Additionally, it is plausible that these diseases influence psychological factors in patients, thereby disrupting normal sleep patterns.

The prevalence of hyperlipidemia among patients with chronic insomnia was examined. Participants were categorized into two groups based on blood lipid reference values: those with abnormal lipid levels and those with normal lipid levels. Comparative analyses were conducted between these groups concerning triglycerides (TG), total cholesterol (TC), high-density lipoprotein cholesterol (HDL-C), and low-density lipoprotein cholesterol (LDL-C), revealing statistically significant differences. Notably, there were no statistically significant differences in age or the proportion of males between the groups. However, a statistically significant difference was observed in body mass index (BMI), with the abnormal lipid group exhibiting a higher BMI than the normal lipid group. This finding aligns with existing research, which indicates that an elevated BMI is more likely to result in lipid abnormalities (22).

Notably, our study revealed that the middle-aged group (45-59 years old) exhibited the highest prevalence of dyslipidemia among patients with chronic insomnia, a finding that warrants further elaboration on its underlying mechanisms. From a pathophysiological perspective, middle age is a critical period characterized by age-related metabolic decline, including reduced insulin sensitivity and altered lipid metabolism, which inherently increase the risk of dyslipidemia (23). Concurrently, chronic insomnia in this age group may exacerbate metabolic disturbances through the dysregulation of neuroendocrine pathways, prolonged sleep deprivation stimulates the secretion of stress hormones such as cortisol and catecholamines, which promote lipolysis and hepatic lipid synthesis, thereby elevating triglyceride and low-density lipoprotein cholesterol levels (24). Lifestyle factors also contribute synergistically, middle-aged adults often face increased work-related stress, irregular dietary patterns (e.g., high-fat, high-sugar diets), and reduced physical activity—all of which are established risk factors for both insomnia and dyslipidemia. Clinically, this comorbidity implies that middle-aged patients with chronic insomnia may require integrated interventions targeting both sleep improvement and lipid management to mitigate long-term cardiovascular risks.

Simultaneously, a comparative analysis of hemoglobin and fasting blood glucose levels was conducted. Among individuals under 45 years of age, hemoglobin levels were within the normal range, yet higher than those observed in all other age groups. This suggests that younger individuals exhibit enhanced adaptability in nutrient absorption and metabolic processes. Furthermore, the analysis of fasting blood glucose levels demonstrated that all age groups exhibited values exceeding the normal range. For individuals under 74 years of age, fasting blood glucose levels progressively increased with age, whereas a declining trend was observed in individuals over 75 years of age. This trend is associated with age-related changes in blood glucose regulation, as existing literature suggests that aging is accompanied by decreased insulin secretion and sensitivity, alterations in metabolic rate, and hormonal fluctuations (25, 26). In individuals over 75 years of age, the observed reduction in blood glucose levels may be attributed to improved glycemic control.

The aforementioned single-center retrospective study examined the prevalence of chronic insomnia among hospitalized patients. However, it is subject to several limitations: (1) The retrospective analysis of data from a single center introduces bias due to regional, disease spectrum, and treatment characteristics, thereby limiting the generalizability of the findings to a broader population; (2) The rate of case accumulation in a single center is slow, resulting in small sample sizes for certain age groups, which diminishes the statistical power of the study; (3)As this study is a retrospective descriptive study, based on the discharge diagnoses collected from our hospital’s electronic medical record system, the majority of the data entered in these forms are highly likely to be patients with secondary insomnia. Therefore, no distinction was made between primary insomnia and secondary insomnia. Further in-depth analysis is needed in the future; (4) we did not include whether the patients with chronic insomnia had any comorbidities such as anxiety, depression, and sleep apnea. These indicators are difficult to extract and quantify in the electronic medical record system, and further supplementation and optimization are needed in the subsequent research.(5) Retrospective studies lack the ability to predefine variable collection protocols, potentially leading to the omission of significant confounding factors and consequently distorting causal inferences. While this study has identified several intriguing phenomena as a preliminary exploratory investigation, further validation through multi-center, prospective cohort studies is necessary.

## Conclusion

In conclusion, chronic insomnia among hospitalized patients deserves increased attention, as it is more prevalent in elderly women. Chronic insomnia was found to coexist with infections, cardiovascular and cerebrovascular diseases, tumors, abnormal blood lipid levels, and elevated fasting blood glucose.

## Data Availability

The original contributions presented in the study are included in the article/supplementary material. Further inquiries can be directed to the corresponding authors.
